# Anemia in women of reproductive age in Ecuador: Data from a national survey

**DOI:** 10.1371/journal.pone.0239585

**Published:** 2020-09-24

**Authors:** Andrea Sosa-Moreno, Sharon Reinoso-González, Miguel Angel Mendez

**Affiliations:** 1 Department of Epidemiology, School of Public Health, University of Michigan, Ann Arbor, MI, United States of America; 2 Grupo de Química Computacional y Teórica, Departamento de Ingeniería Química, Colegio de Ciencias e Ingenierías, Politécnico, Universidad San Francisco de Quito, Quito, Ecuador; University of Georgia, UNITED STATES

## Abstract

**Background:**

Anemia is a condition characterized by a decline in the number of functional red blood cells or hemoglobin. Women of reproductive age from low- and middle-income countries are at higher risk of anemia, which could lead to prenatal, obstetric and perinatal complications. The aim of our study was to explore associations between anemia status and a set of demographic, socio-economic and reproductive factors, among Ecuadorian women of reproductive age (WRA).

**Methods:**

We used data from non-pregnant, WRA (≥12 and ≤49 years) women enrolled in the nationally representative cross-sectional Ecuadorian National Health and Nutrition Survey 2012 (ENSANUT-ECU 2012). Anemia and moderate-severe anemia were assessed using hemoglobin concentrations cutoffs of <12 g/dL and <11 g/dL, respectively. Logistic regression was used to obtain unadjusted and adjusted prevalence odds ratios (aOR). All analyzes were adjusted for multi-stage sampling, stratification and clustering.

**Results:**

The study population included a subset of 7415 non-pregnant WRA. Mean hemoglobin concentration was 12.84 g/dL (95% CI = 12.8–12.9). The overall prevalence of anemia and moderate-severe anemia was 16.8% and 5.0%, respectively. Some factors were associated with an increase in anemia prevalence odds: living in Guayaquil (aOR 1.82, 95% CI 1.16–2.84) and Quito (aOR 1.84, 95% CI 1.17–2.90) compared to living in the rural Amazon, having given birth to more than four alive children compared with being nulliparous (aOR 1.85, 95% CI 1.00–3.43), currently taking contraceptives compared with former use (aOR 1.46, 95% CI 1.09–1.97). In addition, moderate-severe anemia was associated with age and region of residence.

**Conclusion:**

In 2012, the prevalence of anemia among Ecuadorian WRA was considered a mild public health concern. However, we identified groups with higher anemia prevalence. Thus, emphasizing the importance of analyzing the prevalence in sub-populations of WRA and identifying populations where more frequent surveillance may be helpful.

## Introduction

Anemia is a condition characterized by a reduction in the number of functional red blood cells or hemoglobin, the protein responsible for oxygen transportation [1, 2]. Iron deficiency is the most common cause of anemia; however, the causes can be multifactorial, such as parasitic infestations, malaria, inflammation, hemoglobinopathies, as well as renal disease [3–6]. A hemoglobin concentration measurement is part of a routine checkup to assess for anemia, especially in countries with high prevalence [7].

Anemia is a particular public health problem among women 15–49 years year of age in low- and middle-income countries. The World Health Organization (WHO) reported 496 million non-pregnant women with anemia worldwide in 2011, with an increasing trend from 1995 [8–10]. Among this group, the prevalence of anemia was 32.8% worldwide in 2016 [11]. Some of the reasons for the high prevalence of anemia in women include low iron content diet, loss of iron through menstruation and menorrhagia, weight-loss diets, miscarriage, and placental abruption; additionally, anemia is often associated with socioeconomic factors such as education [12–14]. Anemic pregnant women have a higher risk of adverse pregnancy outcomes such as low birth weight, preterm birth, perinatal and neonatal mortality [1, 15]. Other consequences include fatigue and low performance, such as decreased productivity at work [16]. Anemia is considered a mild public health problem among women of reproductive age (WRA: ≥ 12 and < 49 years according to the ENSANUT-ECU 2012 definition) in Ecuador [17]. Despite this, anemia research has focused on children, pregnant women, and the elderly. Studies focusing on WRA typically have a limited number of participants or are focused on a specific community, region, city, or area [18].

Previous anemia estimates for Ecuador have been calculated using data from the Ecuadorian National Health and Nutrition Survey, ENSANUT-ECU 2012, a national and regional representative survey aimed at describing the health and nutrition status of Ecuadorians by collecting information about micronutrients status, maternal and child health, reproductive health and chronic diseases, among others. ENSANUT-ECU 2012 reported a prevalence of anemia of 15.1% among non-pregnant WRA (≥12 and ≤ 49 years) without adjusting for smoking status [19], while non-pregnant women 20 to 49 years of age had a prevalence of 16.9% [20]. More recent data from the World Bank showed that 19% of Ecuadorian women 15 to 49 years old had anemia in 2016 [11]. Anemia is also a mild public health concern in other Latin American countries such as Argentina, Colombia, Costa Rica, Honduras, Nicaragua, Mexico and Peru. In Latin America and the Caribbean, using data between 2000 to 2010, only Chile, Colombia, El Salvador, Costa Rica and Nicaragua had lower anemia prevalence than Ecuador [17]. Exploration of variables associated with anemia status has been limited to economic quintile and ethnicity groups. Additionally, previous estimates were focused on the presence of any anemia, making no distinction between anemia and moderate-severe anemia [19].

Preventive interventions for anemia in WRA can make a positive impact on maternal and infant mortality and morbidity [21]. The present study explores the association between the presence of anemia or moderate-severe anemia with demographic, socio-economic and reproductive factors among non-pregnant, reproductive-aged (≥12 and ≤49 years) women enrolled in the cross-sectional, population-based survey ENSANUT-ECU 2012.

## Methods and materials

### Study population and data collection

Ecuador is a Spanish-speaking country located in northwestern South America. In 2010, the latest National Census estimated a population of 14.483.499 individuals [22]. More recent data estimates a population of 17.4 million people as of March 2020 [23]. Ecuador has a superficial area of 283,561 km^2^ divided into 4 regions: the Coast, the Highlands, the Amazon and the Galapagos Islands. Its currency has been the US dollar since 2000 (official dollarization), and its main exports are petroleum, bananas, cut flowers, and shrimp [24].

We used data from ENSANUT-ECU 2012, a nationally representative cross-sectional survey, that collected information from 0 to 59-year-old individuals throughout the country using 12 different questionnaires (S1 Table). The data reported in ENSANUT-ECU 2012 was collected between 2011 and 2013 by the Ministerio de Salud Pública del Ecuador (MSP) in a joint effort with the Instituto Nacional de Estadística y Censos del Ecuador (INEC). The ENSANUT-ECU 2012 multi-stage sample was stratified by geographical area (urban/rural) and clustered by census tracts. Expansion factors were calculated using the population from the 2010 National Census. A WRA and one subject per each age group were randomly selected within sampled households. Additional data regarding ENSANUT-ECU 2012 methodology can be found elsewhere [19]. Non-identifiable data from the ENSANUT-ECU 2012 is publicly available from the INEC website [25].

**Fig 1:**
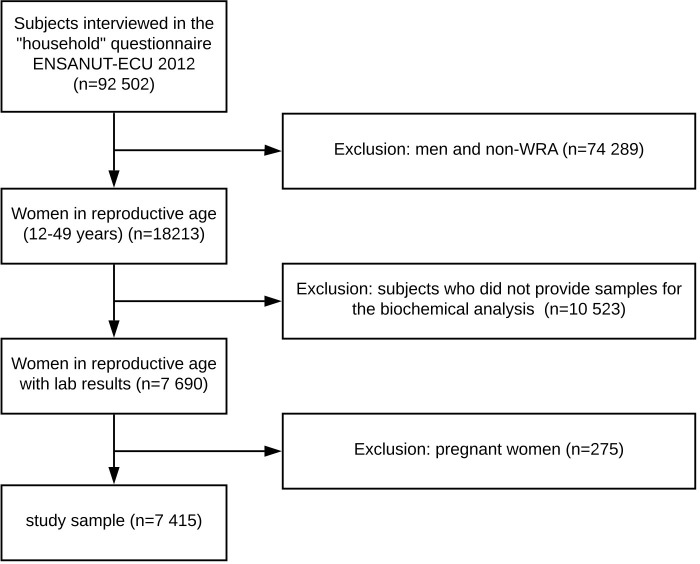
Flowchart of study selection process.

WRA who were included in the household questionnaire, and who completed the women in reproductive age questionnaire, anthropometric measurements and biochemical analysis of the ENSANUT-ECU 2012 were eligible for inclusion into this study (n = 7690). As part of the biochemical analysis, blood samples were collected in a subsample of the households and among subjects who provided consent. Hemoglobin concentrations were assessed by Synlab-Ecuador (formerly Netlab) using an automated blood cell counter. Pregnant women (n = 275) were excluded from the analysis because the mechanisms that lead to anemia among this population may differ from non-pregnant women. The following results are based on a subset of 7415 non-pregnant WRA (Fig 1).

### Anemia assessment

Hemoglobin concentrations were adjusted for altitude and smoking status according to the CDC recommendations [26]. We categorized subjects into those with presence or absence of any anemia (cutoff <12 g/dL) as our primary outcome. In addition, we assessed presence or absence of moderate-severe anemia (cutoff < 11 g/dL). Cutoffs were selected following WHO recommendations for WRA [27].

### Anemia predictors

We studied the association between anemia status and a set of variables, which were chosen based on previous literature describing anemia risk factors [28–32]. Demographic variables included age (12–24, 25–34, 35–49 years), marital status (never, currently and formerly married), education level (none, primary, secondary, higher), region of residence (Urban Highlands, rural Highlands, urban Coast, rural Coast, urban Amazon, rural Amazon, Galapagos, Quito and Guayaquil), and self-reported ethnicity (Indigenous, Afro-Ecuadorian, Montubio, other). Socioeconomic variables included economic quintiles (poorest, poorer, middle, richer, richest), employment status (employed, unemployed), and literacy status (literate, illiterate). Other variables included the number of children born alive (0, 1–2, 3–4, >4), history of adverse pregnancy outcome including stillbirths, miscarriage or abortion (no, yes) and contraceptive history (never, currently, formerly). Our variable assessing contraceptive history included methods, such as sterilization, implants, contraceptive injections, contraceptive pills, copper intrauterine devices (IUDs) and condoms. Up to three measures of height and weight were available per subject. Body Mass Index (BMI) was calculated using the mean height and mean weight per individual. Women were categorized as normal or underweight (lower than 25 kg/m^2^), overweight (25–29.9 kg/m^2^), or obese (greater than or equal than 30 kg/m^2^).

### Statistical analysis

All analyses were corrected for the multi-stage sampling, stratification and clustering as recommended in the ENSANUT-ECU 2012 report [19]. Descriptive statistics were calculated. Weighted proportions were reported using sample weights to obtain nationally representative estimates. Bivariate logistic regression analysis was performed to assess the association between the presence of anemia and each variable without adjusting for other variables. Variables with p-values <0.2 were added into the multivariable model. Multivariable logistic regression measured the association between the prevalence of anemia and each variable while adjusting for the other variables in the model. Statistical analyses were performed in SAS 9.4 (SAS. Cary, North Carolina).

## Results

Among our sample of 7415 WRA, the mean hemoglobin concentration was 12.84 g/dL (95% CI = 12.8–12.9). The overall prevalence of anemia using the 12 g/dL cutoff was 16.8%, while the prevalence of moderate-severe anemia was 5.0%. Table 1 shows the weighted proportion of individuals with respect to all variables analyzed in the present study. Overall, we found an above-average prevalence of anemia and moderate-severe anemia among women in the 35 to 49 years old category (anemia = 17.6%, moderate-severe anemia = 6.4%), formerly married (18.9%, 6.4%), not educated (25.8%, 8.6%), living on the urban coast (18.4%, 5.5%), living in Guayaquil (21.3%, 8.6%), having given birth to 3–4 children (17.4%, 5.9%), Afro-Ecuadorian ethnicity (22.3%, 6.6%), illiterate (23.8%, 8.7%), and normal or underweight (17.1%, 5.2%). Prevalence of anemia was also higher in women living in Quito (19.5%), whereas prevalence of moderate-severe anemia was higher in women living on the rural coast (5.5%), from the poorest quintile (5.5%), and within the Montubio ethnicity (5.8%).

**Table 1:** Anemia (Hb < 12g/dL) and moderate-severe anemia (Hb < 11g/dL) distribution among non-pregnant WRA, ENSANUT-ECU 2012.

			Anemia status		Moderate/severe anemia status	
		Total	< 12 g/dL (n = 1135)	≥ 12 g/dL (ref) (n = 6280)	< 11 g/dL (n = 350)	≥ 11 g/dL (ref) (n = 7065)
		(n = 7415)	N (Wt %)	N (Wt %)	N (Wt %)	N (Wt %)
**Age (years)**						
	12–24	1953	262 (31)	1691 (41)	75 (23)	1878 (40)
	25–34	2644	375 (28)	2269 (27)	95 (36)	2549 (27)
	35–49	2818	498 (41)	2320 (32)	180 (51)	2638 (33)
**Marital status**						
	Never	1452	196 (32)	1256 (43)	54 (26)	1398 (42)
	Currently	5078	772 (57)	4306 (50)	239 (61)	4839 (50)
	Formerly	885	167 (11)	718 (7)	57 (13)	828 (8)
**Education level**						
	None	163	42 (7)	121 (3)	14 (6)	149 (4)
	Primary	2861	430 (37)	2431 (40)	143 (46)	2718 (39)
	Secondary	3085	479 (37)	2606 (36)	149 (33)	2936 (37)
	Higher	1306	184 (19)	1122 (21)	44 (14)	1262 (20)
**Region**						
	Urban Highlands	1661	252 (14)	1409 (16)	78 (14)	1583 (16)
	Rural Highlands	1415	222 (15)	1193 (17)	60 (11)	1355 (17)
	Urban Coast	1108	204 (20)	904 (19)	61 (20)	1047 (19)
	Rural Coast	454	70 (8)	384 (9)	25 (9)	429 (9)
	Urban Amazon	842	92 (2)	750 (2)	28 (2)	814 (2)
	Rural Amazon	928	104 (2)	824 (3)	39 (3)	889 (3)
	Galapagos	221	31 (0)	190 (0)	10 (0)	221 (0)
	Quito	439	86 (19)	353 (17)	19 (12)	420 (17)
	Guayaquil	347	74 (20)	273 (17)	30 (29)	317 (17)
**Economic Quintile**						
	Poorest	1844	293 (21)	1551 (20)	102 (25)	1742 (19)
	Poorer	1687	229 (16)	1458 (20)	61 (15)	1626 (20)
	Middle	1471	236 (22)	1235 (18)	75 (24)	1396 (19)
	Richer	1302	193 (20)	1109 (20)	59 (19)	1243 (20)
	Richest	1110	184 (21)	926 (22)	53 (17)	1057 (22)
**Number of children born alive**						
	0	995	124 (23)	871 (34)	33 (17)	962 (33)
	1–2	3355	478 (36)	2877 (37)	144 (38)	3211 (37)
	3–4	2162	376 (27)	1786 (20)	127 (30)	2035 (21)
	>4	903	157 (14)	746 (9)	46 (15)	857 (9)
**Adverse pregnancy outcome history**						
	No	5791	880 (82)	4911 (82)	268 (83)	5523 (82)
	Yes	1624	255 (18)	1369 (18)	82 (17)	1542 (18)
**Ethnicity**						
	Other	6083	917 (82)	5166 (84)	287 (81)	5796 (84)
	Indigenous	834	122 (9)	712 (7)	32 (8)	802 (7)
	Afro-Ecuadorian	274	61 (4)	213 (4)	18 (3)	256 (4)
	Montubio	224	35 (5)	189 (5)	13 (8)	211 (5)
**Contraceptive History**						
	Never	1188	184 (24)	1004 (32)	51 (21)	1137 (31)
	Currently	5065	788 (63)	4277 (52)	239 (62)	4826 (53)
	Formerly	1162	163 (14)	999 (17)	60 (17)	1102 (16)
**Laboral status**						
	Employed	3259	520 (45)	2739 (40)	154 (43)	3105 (41)
	Unemployed	4156	615 (55)	3541 (60)	196 (57)	3960 (59)
**Literacy status**						
	Literate	7243	1094 (92)	6149 (97)	335 (91)	6908 (96)
	illiterate	172	41 (8)	131 (3)	15 (9)	157 (4)
**BMI (kg/m**^**2**^**)**						
	<25	2914	497 (48)	2417 (48)	151 (44)	2763 (48)
	25.0–29.9	2849	411 (33)	2438 (33)	115 (30)	2734 (33)
	≥30	1652	227 (19)	1425 (19)	84 (26)	1568 (19)

Wt% = weighted percentage corrected for multi-stage sampling, stratification and clustering.

Unadjusted associations between anemia status and all variables, except for adverse pregnancy outcome history, ethnicity and BMI, had p-values lower than 0.2 and therefore were added into the multivariable model. Results from the multivariable logistic regression models showed at least one category of marital status, region of residence, number of children born alive, and contraceptive history associated with anemia status (Overall effect p-value). After adjusting for covariates, currently married women had lower prevalence odds of anemia compared with formerly married women (aOR = 0.67, 95% CI: 0.51–0.88). Women living in Quito (aOR = 1.84, 95% CI: 1.17–2.90), Guayaquil (aOR = 1.82, 95% CI: 1.16–2.84) and on the urban Coast (aOR = 1.53, 95% CI: 0.97–2.42) had a higher prevalence odds of anemia compared with women living in the rural Amazon. Also, women with more than four children born alive had higher prevalence odds of anemia (aOR = 1.85, 95% CI: 1.00–3.43) compared with nulliparous women. Women currently taking contraceptives had a higher prevalence odds of anemia compared with former contraceptive users (aOR = 1.46, 95% CI: 1.09–1.97) (Table 2). Other factors were not associated with anemia status.

**Table 2:** Unadjusted and adjusted odds ratios between anemia (hb < 12g/dL) and studied variables.

		Unadjusted analysis			Adjusted analysis		
		Odds ratio	T test	Overall effect p-value	Odds ratio	T test	Overall effect p-value
		95% CI	p-value		95% CI	p-value	
**Age (years)**							
	12–24 (Ref)	---	---	<0.0001*	---	---	0.792
	25–34	1.35 (1.04–1.75)	0.024*		0.98 (0.74–1.28)	0.851	
	35–49	1.66 (1.31–2.10)	<0.0001*		1.03 (0.73–1.45)	0.867	
**Marital status**							
	Never	0.49 (0.35–0.69)	<0.0001*	0.0004*	0.71 (0.47–1.07)	0.099	0.015*
	Currently	0.76 (0.59–0.98)	0.037*		0.67 (0.51–0.88)	0.004*	
	Formerly (Ref)	---	---		---	---	
**Education level**							
	None (Ref)	---	---	0.051*	---	---	0.915
	Primary	0.46 (0.26–0.82)	0.009*		0.89 (0.33–2.43)	0.824	
	Secondary	0.51 (0.30–0.89)	0.018*		0.96 (0.33–2.72)	0.932	
	Higher	0.45 (0.25–0.80)	0.008*		0.90 (0.30–2.71)	0.847	
**Region**							
	Urban Highlands	1.32 (0.86–2.03)	0.194	<0.0001*	1.40 (0.90–2.18)	0.133	0.005*
	Rural Highlands	1.46 (0.85–2.49)	0.167		1.44 (0.84–2.47)	0.174	
	Urban Coast	1.61 (1.05–2.49)	0.031*		1.53 (0.97–2.42)	0.067	
	Rural Coast	1.39 (0.86–2.24)	0.173		1.31 (0.80–2.13)	0.278	
	Urban Amazon	1.10 (0.66–1.83)	0.706		1.10 (0.66–1.81)	0.718	
	Rural Amazon (Ref)	---	---		---	---	
	Galapagos	1.83 (1.20–2.81)	0.989		1.06 (0.60–1.88)	0.829	
	Quito	1.83 (1.15–2.91)	0.012*		1.84 (1.17–2.90)	0.009*	
	Guayaquil	1.83 (1.20–2.81)	0.006*		1.82 (1.16–2.84)	0.009*	
**Economic Quintile**							
	Poorest	1.10 (0.82–1.48)	0.533	0.077	1.12 (0.81–1.56)	0.494	0.138
	Poorer	0.83 (0.64–1.07)	0.145		0.86 (0.64–1.15)	0.299	
	Middle	1.28 (1.00–1.63)	0.047*		1.26 (0.96–1.63)	0.070	
	Richer	1.06 (0.69–1.62)	0.795		1.08 (0.71–1.65)	0.716	
	Richest (Ref)	---	---		---	---	
**Number of children born alive**							
	0 (Ref)	---	---	<0.0001*	---	---	0.026*
	1–2	1.40 (0.89–2.20)	0.139		1.19 (0.69–2.05)	0.536	
	3–4	1.89 (1.28–2.78)	0.002*		1.55 (0.88–2.72)	0.127	
	>4	2.30 (1.45–3.62)	0.0005*		1.85 (1.00–3.43)	0.052	
**Adverse pregnancy outcome history**							
	No (Ref)	---		0.775			
	Yes	1.04 (0.79–1.37)	0.775				
**Ethnicity**							
	Other (Ref)	---	---	0.226			
	Indigenous	1.36 (0.97–1.92)	0.077				
	Afro-Ecuadorian	1.22 (0.86–1.73)	0.259				
	Montubio	0.98 (0.61–1.57)	0.916				
**Contraceptive History**							
	Never	0.93 (0.62–1.39)	0.728	0.005*	1.25 (0.78–2.01)	0.349	0.025*
	Currently	1.50 (1.12–2.00)	0.007*		1.46 (1.09–1.97)	0.013*	
	Formerly (Ref)	---	---		---	---	
**Employment status**							
	Employed (Ref)	---	---	0.181	---	---	0.606
	Unemployed	0.822 (0.62–1.10)	0.181		0.93 (0.71–1.22)	0.606	
**Literacy status**							
	Literate (Ref)	---	---	<0.0001*	---	---	0.174
	illiterate	2.31 (1.56–3.42)	<0.0001*		1.75 (0.78–3.91)	0.174	
**Body Mass Index (kg/m**^**2**^**)**							
	Normal or Underweight <25 (Ref)	---	---	0.992			
	Overweight 25.0–29.9	1.02 (0.80–1.29)	0.903				
	Obese ≥30	1.01 (0.77–1.34)	0.935				

Multivariable logistic regression adjusted for age, marital status, education level, region of residence, economic quintile, number of children born alive, contraceptive history, employment status and literacy status.

Missing Data (Analytic Sample) Economic Quintile (N = 1).

* p value ≤ 0.05.

Regarding moderate-severe anemia, results are summarized in Table 3. From the univariate analysis, we found all variables, except for history of adverse pregnancy outcomes, ethnicity and employment status, had p-values lower than 0.2 and therefore they were included in the multivariable analysis. Results from the multivariable logistic regression models showed that at least one category of region of residence was associated with moderate-severe anemia (Overall effect p-value). After adjusting for covariates, we found that the prevalence odds of anemia increased with age. Women 35 to 49 years of age had higher prevalence odds of moderate-severe anemia than women 12 to 24 years old (aOR = 2.32, 95% CI: 1.09–4.94). Point estimates also showed a decrease in prevalence of moderate-severe anemia within higher levels of education, but this association was not statistically significant. Women who were living in the urban highlands (aOR = 1.70, 95% CI: 1.00–2.90), urban coast (aOR = 1.76, 95% CI: 0.96–3.23), and Guayaquil (3.11, 95% CI: 1.88–5.13) had higher prevalence odds of moderate-severe anemia compared with women living in the rural highlands. Overweight women had lower anemia prevalence odds compared with normal or underweight women (aOR = 0.68, 95% CI: 0.46–1.00).

**Table 3:** Unadjusted and adjusted odds ratios between moderate-severe anemia (hb < 11g/dL) and studied variables.

		Unadjusted analysis			Adjusted analysis		
		Odds ratio	T test	Overall effect	Odds ratio	T test	Overall effect
		95% CI	p-value	p-value	95% CI	p-value	p-value
**Age (years)**							
	12–24 (Ref)	---	---	<0.0001*	---	---	0.061
	25–34	1.70 (1.09–2.64)	0.021*		1.44 (0.81–2.56)	0.207	
	35–49	2.76 (1.75–4.37)	<0.0001*		2.32 (1.09–4.94)	0.030*	
**Marital status**							
	Never (Ref)	---	---	0.008*	---	---	0.407
	Currently	1.95 (1.15–3.28)	0.013*		1.00 (0.58–1.73)	0.999	
	Formerly	2.65 (1.44–4.89)	0.002*		1.39 (0.72–2.67)	0.318	
**Education level**							
	None (Ref)	---	---	0.089	---	---	0.104
	Primary	0.45 (0.21–0.96)	0.040*		2.13 (0.52–8.66)	0.287	
	Secondary	0.769 (0.34–1.75)	0.528		1.48 (0.35–6.24)	0.593	
	Higher	0.58 (0.24–1.38)	0.213		1.18 (0.32–4.33)	0.801	
**Region**							
	Urban highlands	1.31 (0.75–2.29)	0.976	0.0003*	1.70 (1.00–2.90)	0.050*	<0.0001*
	Rural highlands (Ref)	---	0.477		---	---	
	Urban coast	1.58 (0.91–2.73)	0.688		1.76 (0.96–3.23)	0.066	
	Rural coast	1.50 (0.79–2.83)	0.824		1.50 (0.78–2.86)	0.219	
	Urban Amazon	1.28 (0.64–2.55)	0.945		1.54 (0.78–3.07)	0.213	
	Rural Amazon	1.67 (0.85–3.30)	0.669		1.80 (0.87–3.71)	0.109	
	Galapagos	1.33 (0.60–2.91)	---		1.87 (0.93–3.79)	0.080	
	Quito	1.04 (0.47–2.29)	0.686		1.31 (0.49–3.47)	0.583	
	Guayaquil	2.58 (1.67–3.98)	0.147		3.11 (1.88–5.133)	<0.0001*	
**Economic Quintile**							
	Poorest	1.72 (0.99–2.99)	0.053	0.015*	1.57 (0.76–3.30)	0.220	0.138
	Poorer	1.04 (0.49–2.24)	0.914		0.90 (0.39–2.04)	0.789	
	Middle	1.76 (1.18–2.63)	0.006*		1.46 (0.88–2.43)	0.140	
	Richer	1.25 (0.70–2.24)	0.449		1.10 (0.66–1.84)	0.703	
	Richest (Ref)	---	---		---	---	
**Number of children born alive**							
	0 (Ref)	---	---	<0.004*	---	---	0.230
	1–2	2.01 (1.20–3.37)	0.008*		1.73 (0.95–3.15)	0.075	
	3–4	2.81 (1.54–5.14)	0.001*		1.82 (0.81–4.08)	0.148	
	>4	3.06 (1.52–6.16)	0.002*		1.57 (0.49–5.08)	0.447	
**Adverse pregnancy outcome history**							
	No (Ref)	---		0.838			
	Yes	0.96 (0.63–1.46)	0.838				
**Ethnicity**							
	Other (Ref)	---	---	0.584			
	Indigenous	1.27 (0.61–2.62)	0.520				
	Afroecuadorian	0.95 (0.53–1.72)	0.871				
	Montubio	1.52 (0.70–3.28)	0.284				
**Contraceptive History**							
	Never (Ref)	---	---	0.073	---	---	0.846
	Currently	1.71 (1.06–2.78)	0.029*		0.87 (0.50–1.52)	0.620	
	Formerly	1.51 (0.96–2.37)	0.077		0.85 (0.49–1.49)	0.575	
**Employment status**							
	Employed (Ref)	---	---	0.639			
	Unemployed	0.91 (0.60–1.38)	0.639				
**Literacy status**							
	Literate (Ref)	---	---	0.002*	---	---	0.098
	illiterate	2.37 (1.39–4.03)	0.002*		2.19 (0.86–5.58)	0.098	
**Body Mass Index (kg/m**^**2**^**)**							
	Normal or Underweight <25 (Ref)	---	---	0.193	---	---	0.148
	Overweight 25.0–29.9	1.48 (0.97–2.27)	0.070		0.68 (0.46–1.00)	0.052	
	Obese ≥30	0.99 (0.68–1.46)	0.969		0.88 (0.51–1.52)	0.631	

Multivariable logistic regression adjusted for age, marital status, education level, region of residence, economic quintile, number of children born alive, contraceptive history, literacy status and body mass index.

Missing Data (Analytic Sample) Economic Quintile (N = 1).

* p value ≤ 0.05.

## Discussion

The present study has analyzed the association between anemia prevalence and a set of demographic, socio-economic and reproductive factors among Ecuadorian non-pregnant WRA with data available from the ENSANUT-ECU 2012. Additionally, we analyzed moderate-severe anemia status, an important assessment since moderate-severe anemia has additional serious health consequences [33]. The results of the multivariable analysis showed that living on the urban coast, Quito and Guayaquil, having given birth to more than four children, and currently using contraceptive methods were associated with higher prevalence odds of anemia, whereas being currently married was associated with lower prevalence odds. In addition, women 35 to 49 years of age, living in the urban highlands, urban coast, and Guayaquil, and being overweight was associated with a higher prevalence odds of moderate-severe anemia.

We found that among non-pregnant Ecuadorian WRA, the overall prevalence of anemia and moderate-severe anemia was 16.8% and 5%, respectively. According to WHO, this proportion of anemia is in the upper end of the “mild public health concern” category (5%-19.9%) [27]. Ecuadorian women have a lower prevalence of anemia compared to global estimates (roughly 30%, non-pregnant in 2011) [8] and estimates from Latin America and the Caribbean (22.5%, non-pregnant women 15 to 49 years old) and South America alone (24.2%, non-pregnant women 15 to 49 years old) [34]. The prevalence of anemia in Mexico/Central America (16.3%) is close to that of Ecuador [34]. Nepal, another developing country, reported a 41% anemia prevalence among 15–49 year old women [32].

Regarding moderate-severe anemia, the prevalence among 15–49 year old was 7% and 0.3% among Nepalese women [32]. In contrast, countries such as the United States reported a moderate-severe anemia prevalence of 2.5% [33], half the prevalence reported in this study. Differences in prevalence estimates can be explained by variation in the definition of reproductive age among studies and differences in diet, health and nutrition programs among countries.

The prevalence of anemia and moderate-severe anemia increased with age in the non-adjusted model. This trend was consistent only with the multivariate analysis of moderate-severe anemia where women aged 35–49 years were more likely to have moderate-severe anemia compared with the youngest group. Other studies using data from developing and developed countries are consistent with our results [32, 35]. Gautam et al., found no significant association between anemia status and women’s age [32], while Le et al., found that anemia-age trends follow a bimodal distribution in the risk of anemia in women with a peak at 40–49 years [33]. Regarding moderate-severe anemia, it is possible that some health conditions, such as iron deficiency, hypoalbuminemia, cancer and chronic diseases, increase the prevalence in older adults in Ecuador [36]. Additionally, women in the 35–49 category may include subjects who are experiencing perimenopause, with symptoms such as menorrhagia (heavy menstrual bleeding), which increases the risk of anemia [37].

In our study, currently and never married women had lower prevalence odds of anemia compared with formerly married women even after controlling for other factors. In lower income countries, married and formerly married women are likely to carry out most activities including work on crops, domestic activities and taking care of children. Having so many responsibilities on their shoulders could harm their health status [13]. Although not statistically significant, the higher the level of education, the lower the prevalence odds of anemia. This could be explained by the fact that educated women would receive higher incomes and therefore eat a balanced diet including more meat or other iron-containing food [38].

Region of residence was associated with both anemia and moderate-severe anemia. We reported some urban settings having higher prevalence odds of anemia and moderate-severe anemia than similar rural settings. Guayaquil had a population of around 2,300,000 inhabitants in 2010 [22], and it was the most populated city in Ecuador at the time of the ENSANUT-ECU 2012 survey. Prior to 1990, Cañizares et al. indicated that the prevalence of anemia in Ecuador differed markedly according to the areas of the country, which partially agrees with our study [39]. Other studies also reported significant variation in anemia prevalence across geographical regions [40]. According to Elzahaf and Omar, the variation in the prevalence of anemia across regions, provinces and cities may be due to characteristics and nutritional habits of the population [41]. Information about additional factors such as genetic traits, some non-communicable diseases, human immunodeficiency virus (HIV) infections, and inadequate bioavailable dietary iron, folic acid or vitamin B12, among others [10] are needed to explain regional differences.

We could not draw solid conclusions about the association between economic quintile and anemia status consistent with another study from Nepal [32]. Furthermore, the fact that women within the middle quintile had a higher prevalence compared with the richest quintile, seems to show no clear trend. In contrast, an analysis of demographic and health surveys in 32 countries with low and middle income reported that women who belonged to the lowest quintile had a 25% higher risk of developing anemia compared with women who belonged to the highest wealth quintile [42]. It is worth noticing that in Ecuador the incidence of poverty has decreased while the Human Development Index has increased in recent years and the country is classified by the World Bank as an upper middle-income country [43, 44]. During 2014, the average price of the Ecuadorian basic family basket of commodities (Canasta básica) was $636.78 [45]. As of June 2017, 5% of Ecuadorians belonged to households with an economic income of up to $153.0 per month, and 22.7% and 23.5% of men and women were considered within the category of poverty respectively and [46]. More recent data showed that by June 2019 the average monthly family income was $735.47, whereas the basic family basket of commodities cost was $715.83. Nevertheless, inequality and poverty persist in some portions of the population, especially in the Highlands and in the Coastal region, both in urban and rural areas [45].

The prevalence of anemia in Ecuadorian women was associated with the number of children born alive: the greater the number of children born alive, the greater the prevalence compared with nulliparous women, in line with other studies [42]. Those results remained constant after adjusting for covariates. A possible explanation for this is the fact that the more children a woman bears, the more nutrients are depleted from her body [47]. During pregnancy, the blood volume increases up to 50% leading to a higher iron demand that could affect anemia prevalence if that demand is not met by the woman’s diet [48]. Repetitive pregnancies also reduce the body's iron stores [49].

Although Afro-Ecuadorians have a higher prevalence of anemia and moderate-severe anemia compared with the national prevalence, ethnicity was not associated with anemia status nor moderate-severe anemia in our multivariable analysis in agreement with previous literature [32]. Among women between 20 to 49 years, Freire, et al. reported that numerically, Afro-Ecuadorian women had a slightly higher prevalence of anemia compared with other ethnicities; however, they did not perform any statistical test [19]. Another study showed that older Afro-Ecuadorian individuals (median age 71.8, SD: 8.2) had a higher prevalence of anemia compared with other ethnicities [36].

Contraceptive history was associated with anemia but not with moderate-severe anemia after controlling for covariates. Women currently using contraceptives had a higher prevalence of anemia compared with women who formerly used contraceptives. In contrast with our results, in the United States, NHANES data (2003–2012) indicated that the use of contraceptives in young women (12–21 years) was associated with decreased odds of iron deficiency anemia [33]. A study conducted in the female population of Tanzania indicated that hormonal contraceptives reduced the likelihood of developing health problems associated with pregnancy, among them iron deficiency [50]. However, both studies targeted different segments of the populations than ours (12-21-year-old and pregnant women in contrast to non-pregnant 12-49-year-old) which could explain our different results. Additionally, we did not make any distinction between contraceptive methods.

Although not statistically significant, illiterate women had a higher prevalence of moderate-severe anemia compared with literate women. Literate women may have a better standard of living and adequate health services reducing the chances of anemia which could partially explain our results. Ecuador had a literacy rate of 93.3% for females aged 15 and above during 2016. Even though the illiteracy rate has dropped in recent years, Ecuador is the 69th country in the ranking of literacy rate [51].

The strength of our study is that our results are based on nationally representative data which allowed us to generate nationally representative anemia prevalence estimates. However, our study also has several limitations. First, there are other variables that have been associated with anemia but were not included in this analysis because those variables were not included in the ENSANUT-ECU 2012 survey. Such variables include: parasitic infection, HIV, and malaria status. Second, we did not consider dietary factors that could be associated with anemia. Finally, ENSANUT-ECU 2012 is a cross-sectional survey where anemia status and variables analyzed in this study were measured at a single time, thus it does not allow us to understand the causal effects of the factors we identified to be associated with anemia in WRA in Ecuador.

## Conclusion

We found an overall prevalence of anemia and moderate-severe anemia of 16.8% and 5%, respectively, among Ecuadorian non-pregnant WRA. This percentage positions anemia as a mild public health concern. Additionally, we explored a set of demographic, socio-economic and reproductive factors associated with anemia and moderate-severe anemia. We found that living in the urban coast, Quito and Guayaquil; having given birth to more than four children; and currently using contraceptives were associated with higher prevalence of anemia. Furthermore, women aged 35–49 years, living in the urban highlands, urban coast or Guayaquil had a higher prevalence of moderate-severe anemia. Thus, emphasizing the importance of analyzing the prevalence in sub-populations of WRA and identifying populations where more frequent surveillance may be helpful. The results of our work will help generate hypotheses about potential risk factors for anemia among these populations. More studies addressing anemia status are needed to understand if women within those groups would benefit from intervention programs targeting anemia.

## Supporting information

S1 Table Data collection surveys used in ENSANUT-ECU 2012. (DOCX)

S1 File (ZIP)

## References

[1] Levy TS, De la Cruz Góngora V, Villalpando S. Anemia: Causes and Prevalence In: Encyclopedia of Food and Health [Internet]. Elsevier; 2016 [cited 2020 Apr 15]. p. 156–63. Available from: https://linkinghub.elsevier.com/retrieve/pii/B9780123849472000295

[2] Meena K, Tayal DK, Gupta V, Fatima A. Using classification techniques for statistical analysis of Anemia. Artificial Intelligence in Medicine. 2019 3;94:138–52. 10.1016/j.artmed.2019.02.005 30871679

[3] Solovyova AV, Gace V, Ermolenko KS, Khorolskiy VA. Anemia in Women of Reproductive Age In: Khan J, editor. Current Topics in Anemia [Internet]. InTech; 2018 [cited 2020 Apr 15]. Available from: http://www.intechopen.com/books/current-topics-in-anemia/anemia-in-women-of-reproductive-age

[4] Percy L, Mansour D, Fraser I. Iron deficiency and iron deficiency anaemia in women. Best Practice & Research Clinical Obstetrics & Gynaecology. 2017 4;40:55–67. 28029503 10.1016/j.bpobgyn.2016.09.007

[5] Percy L, Mansour D. Iron deficiency and iron-deficiency anaemia in women’s health. Obstet Gynecol. 2017 4;19(2):155–61. 10.1016/j.bpobgyn.2016.09.007 28029503

[6] Haidar J. Prevalence of Anaemia, Deficiencies of Iron and Folic Acid and Their Determinants in Ethiopian Women. J Health Popul Nutr. 2010 9 6;28(4):359–68. 10.3329/jhpn.v28i4.6042 20824979 PMC2965327

[7] Freire WB. Hemoglobin as a predictor of response to iron therapy and its use in screening and prevalence estimates. The American Journal of Clinical Nutrition. 1989 12 1;50(6):1442–9. 10.1093/ajcn/50.6.1442 2596434

[8] The global prevalence of anaemia in 2011. [Internet]. Geneva. World Health Organization: WHO; 2015. Available from: https://apps.who.int/iris/bitstream/handle/10665/177094/9789241564960_eng.pdf;jsessionid=7D2817244226D3DBD69DC6FF6F7A582D?sequence=1

[9] Vos T, Allen C, Arora M, Barber RM, Bhutta ZA, Brown A, et al Global, regional, and national incidence, prevalence, and years lived with disability for 310 diseases and injuries, 1990–2015: a systematic analysis for the Global Burden of Disease Study 2015. The Lancet. 2016 10;388(10053):1545–602. 10.1016/S0140-6736(16)31678-6 PMC5055577 27733282

[10] Stevens GA, Finucane MM, De-Regil LM, Paciorek CJ, Flaxman SR, Branca F, et al Global, regional, and national trends in haemoglobin concentration and prevalence of total and severe anaemia in children and pregnant and non-pregnant women for 1995–2011: a systematic analysis of population-representative data. The Lancet Global Health. 2013 7;1(1):e16–25. 10.1016/S2214-109X(13)70001-9 25103581 PMC4547326

[11] The World Bank Group. Prevalence of anemia among women of reproductive age (% of women ages 15–49) [Internet]. [cited 2019 Sep 6]. Available from: https://data.worldbank.org/indicator/SH.ANM.ALLW.ZS

[12] Kusumi E, Shoji M, Endou S, Kishi Y, Shibata T, Murashige N, et al Prevalence of Anemia among Healthy Women in 2 Metropolitan Areas of Japan. International Journal of Hematology. 2006 10 1;84(3):217–9. 10.1532/IJH97.06097 17050194

[13] Adams C, Costello A, Flynn S. Iron deficiency anaemia in Ecuador: does education matter? [Internet]. 2019 Jun. Available from: https://pdfs.semanticscholar.org/cf26/89806194d67bf893d3615f398dc7e8f096db.pdf

[14] Kamruzzaman Md, Rabbani MdG, Saw A, Sayem MdA, Hossain MdG. Differentials in the prevalence of anemia among non-pregnant, ever-married women in Bangladesh: multilevel logistic regression analysis of data from the 2011 Bangladesh Demographic and Health Survey. BMC Women’s Health. 2015 12;15(1):54. 26219633 10.1186/s12905-015-0211-4 PMC4517492

[15] Rahman MM, Abe SK, Rahman MS, Kanda M, Narita S, Bilano V, et al Maternal anemia and risk of adverse birth and health outcomes in low- and middle-income countries: systematic review and meta-analysis1,2. The American Journal of Clinical Nutrition. 2016 2 1;103(2):495–504. 10.3945/ajcn.115.107896 26739036

[16] Haas JD, Brownlie T. Iron deficiency and reduced work capacity: a critical review of the research to determine a causal relationship. The Journal of nutrition. 2011;131(2):676S–690S. 10.1093/jn/131.2.676S 11160598

[17] Mujica-Coopman MF, Brito A, López de Romaña D, Ríos-Castillo I, Cori H, Olivares M. Prevalence of Anemia in Latin America and the Caribbean. Food Nutr Bull. 2015 6;36(2_suppl):S119–28. 26125197 10.1177/0379572115585775

[18] Freire WB, Silva-Jaramillo KM, Ramírez-Luzuriaga MJ, Belmont P, Waters WF. The double burden of undernutrition and excess body weight in Ecuador. The American Journal of Clinical Nutrition. 2014 12 1;100(6):1636S–1643S. 10.3945/ajcn.114.083766 25411306

[19] Freire WB, Ramirez-Luzuriaga MJ, Belmont P, Mendieta MJ, Silva-Jaramillo MK, Romero N, et al Tomo I: Encuesta Nacional de Salud y Nutrición de la población ecuatoriana de cero a 59 años. ENSANUT-ECU 2012. Quito-Ecuador: Ministerio de Salud Pública/Instituto Nacional de Estadísticas y Censos.;

[20] Petry N, Olofin I, Hurrell R, Boy E, Wirth J, Moursi M, et al The Proportion of Anemia Associated with Iron Deficiency in Low, Medium, and High Human Development Index Countries: A Systematic Analysis of National Surveys. Nutrients. 2016 11 2;8(11):693. 10.3390/nu8110693 PMC5133080 27827838

[21] WHO. Global targets 2015. To improve maternal, infant and young child nutrition [Internet]. World Health Organization; Available from: https://www.who.int/nutrition/topics/nutrition_globaltargets2025/en/

[22] INEC. Censo de Poblacion y Vivienda [Internet]. 2010. Available from: http://www.ecuadorencifras.gob.ec/censo-de-poblacion-yvivienda/

[23] INEC. Contador Nacional [Internet]. 2020. Available from: https://www.ecuadorencifras.gob.ec/estadisticas/

[24] The observatory of economic complexity. Ecuador (ECU) Exports, Imports, and Trade Partners [Internet]. Available from: https://atlas.media.mit.edu/en/profile/country/ecu/

[25] Encuesta Nacional de Salud, Salud Reproductiva y Nutrición (ENSANUT)-2012 [Internet]. [cited 2020 Aug 17]. Available from: https://www.ecuadorencifras.gob.ec/encuesta-nacional-de-salud-salud-reproductiva-y-nutricion-ensanut-2012/

[26] CDC. Recommendations to Prevent and Control Iron Deficiency in the United States [Internet]. 1998. Available from: https://www.cdc.gov/mmwr/preview/mmwrhtml/00051880.htm 9563847

[27] WHO. Haemoglobin concentrations for the diagnosis of anaemia and assessment of severity. Vitamin and Mineral Nutrition Information System. [Internet]. Geneva: World Health Organization; 2011. Available from: https://www.who.int/vmnis/indicators/haemoglobin.pdf

[28] Bentley ME, Griffiths PL. The burden of anemia among women in India. Eur J Clin Nutr. 2003 1;57(1):52–60. 10.1038/sj.ejcn.1601504 12548297

[29] Nguyen PH, Gonzalez-Casanova I, Nguyen H, Pham H, Truong TV, Nguyen S, et al Multicausal etiology of anemia among women of reproductive age in Vietnam. Eur J Clin Nutr. 2015 1;69(1):107–13. 10.1038/ejcn.2014.181 25205323

[30] AlQuaiz AM, Gad Mohamed A, Khoja TAM, AlSharif A, Shaikh SA, Al Mane H, et al Prevalence of Anemia and Associated Factors in Child Bearing Age Women in Riyadh, Saudi Arabia. Journal of Nutrition and Metabolism. 2013;2013:1–7. 10.1155/2013/636585 PMC3800602 24205435

[31] UNICEF. The State of the World’s Children. [Internet]. Oxford: University Press: New York, NY; 1998. Available from: http://www.unicef.org/sowc98/pdf.htm

[32] Gautam S, Min H, Kim H, Jeong H-S. Determining factors for the prevalence of anemia in women of reproductive age in Nepal: Evidence from recent national survey data. Kabir R, editor. PLoS ONE. 2019 6 12;14(6):e0218288 10.1371/journal.pone.0218288 31188883 PMC6561639

[33] Le CHH. The Prevalence of Anemia and Moderate-Severe Anemia in the US Population (NHANES 2003–2012). Collins JF, editor. PLoS ONE. 2016 11 15;11(11):e0166635 10.1371/journal.pone.0166635 27846276 PMC5112924

[34] Pan American Health Organization. Anemia in Latin America and the Caribbean, 2009: Situation analysis, trends and implications for public health programming [Internet]. Washington, D.C: PAHO; 2010. Available from: https://www.paho.org/hq/dmdocuments/2011/anemiaLAC.pdf

[35] Wirth JP, Woodruff BA, Aaron G. Predictors of anemia in women of reproductive age: Biomarkers Reflecting Inflammation and Nutritional Determinants of Anemia (BRINDA) project. Am J Clin Nutr. 106. 10.3945/ajcn.116.143073 PMC5490645 28615262

[36] Orces CH. Prevalence of Anemia among Older Adults Residing in the Coastal and Andes Mountains in Ecuador: Results of the SABE Survey. Current Gerontology and Geriatrics Research. 2017;2017:1–10. 10.1155/2017/4928786 PMC5339481 28321252

[37] Firquet A, Kirschner W, Bitzer J. Forty to fifty-five-year-old women and iron deficiency: clinical considerations and quality of life. Gynecological Endocrinology. 2017 7 3;33(7):503–9. 10.1080/09513590.2017.1306736 28347197

[38] Robinson SM, Crozier SR, Borland SE, Hammond J, Barker DJP, Inskip HM. Impact of educational attainment on the quality of young women’s diets. Eur J Clin Nutr. 2004 8;58(8):1174–80. 10.1038/sj.ejcn.1601946 15054431

[39] Canizares C, Bonilla R, Vasquez C. Prevalence of different types of anemia in Ecuador. Braz J Med Biol Res. 1988;21(4):767–72. 3266472

[40] Bora K. Temporal trends and differential patterns in the prevalence of severe anaemia in India: observations from country‐wide haemoglobin determinations 2008–2018. Trop Med Int Health. 2019 5 6;tmi.13240. 10.1111/tmi.13240 31004455

[41] Elzahaf R, Omar M. Prevalence of anaemia among pregnant women in Derna city, Libya. Int J Community Med Public Health. 2016;1915–20.

[42] Balarajan Y, Ramakrishnan U, Özaltin E, Shankar AH, Subramanian S. Anaemia in low-income and middle-income countries. The Lancet. 2011 12;378(9809):2123–35. 10.1016/S0140-6736(10)62304-5 21813172

[43] Roser M. Human Development Index (HDI) [Internet]. 2014. Available from: https://ourworldindata.org/human-development-index

[44] The World Bank Group. Ecuador [Internet]. Available from: https://data.worldbank.org/country/ecuador

[45] INEC. Serie histórica de la canasta familar básica nacional [Internet]. 2019. Available from: https://www.ecuadorencifras.gob.ec/canasta/

[46] Banco Central del Ecuador. Reporte de pobreza, ingreso y desigualdad: Junio 2017 [Internet]. 2019 Jul. Available from: https://contenido.bce.fin.ec/documentos/Estadisticas/SectorReal/Previsiones/IndCoyuntura/Empleo/PobrezaJun2017.pdf

[47] Lopez C, Tates S. Evaluación del uso del índice de fluorescencia de reticulocitos (IRF) y de la carga de hemoglobina del reticulocito (RET-HE) como indicadores de reserva corporal de hierro y de respuesta terapeútica a la suplementación de hierro en mujeres embarazadas [Internet]. 2013. Available from: http://www.dspace.uce.edu.ec/bitstream/25000/1327/1/T-UCE-0006-42.pdf

[48] Sotunde OF, Sanni SA, Onabanjo OO, Olayiwola IO, Agbonlahor M. A retrospective study of the health profile of neonates of mothers with anemia in pregnancy and pregnancy induced hypertension in Lagos, Nigeria. J Public Health Africa [Internet]. 2014 7 4 [cited 2020 Apr 15];5(2). Available from: http://www.publichealthinafrica.org/index.php/jphia/article/view/286 10.4081/jphia.2014.286 PMC5345411 28299124

[49] Mawani M, Aziz Ali S. Iron Deficiency Anemia among Women of Reproductive Age, an Important Public Health Problem: Situation Analysis. Reprod Syst Sex Disord [Internet]. 2016 [cited 2020 Apr 15];5(3). Available from: https://www.omicsonline.org/open-access/iron-deficiency-anemia-among-women-of-reproductive-age-an-important-public-health-problem-situation-analysis-2161-038X-1000187.php?aid=78570

[50] Lokare P, Gattani P, Karanjekar V, Kulkarni A. A study of prevalence of anemia and sociodemographic factors associated with anemia among pregnant women in Aurangabad city, India. Ann Nigerian Med. 2012;6(1):30.

[51] UNESCO. Ecuador: statistics [Internet]. Available from: http://uis.unesco.org/en/country/ec

