# Supplementary material for: Anemia in women of reproductive age in Ecuador: Data from a national survey
Source: PLoS One. 2020 Sep 24;15(9):e0239585. doi: 10.1371/journal.pone.0239585 (PMC7514054; doi:10.1371/journal.pone.0239585)
Supplement: S1 Table — (DOCX) [file pone.0239585.s001.docx]

**S1 Table. Data collection surveys used in ENSANUT-ECU 2012**

| **Questionnaire** | **Description of collected data** | **Number of subjects interviewed** |
| --- | --- | --- |
| Household | Names of all members of a household, household characteristics, socioeconomic status | 92502 |
| Women in reproductive age among women from 12 to 49 years | History of pregnancies and births, maternal and reproductive health | 18213 |
| Breastfeeding in children under 3 years | Characteristics of breastfeeding and supplementary feeding | 5972 |
| Health in children under 5 years | Children’s health condition: immunizations, acute diarrhea, respiratory infections | 10098 |
| Risk factors among children from 5 to 10 years | Life quality, oral health, television and video game habits | 8433 |
| Risk factors among subjects from 10 to 20 years | Food habits, smoking, alcohol drinking, oral health, television and video game habits | 13162 |
| Risk factors among men and women from 20 to 60 years | Food habits, smoking and alcohol drinking, underlying medical conditions. | 30428 |
| Physical activity among subjects from 18 to 60 years | Transportation and recreation habits | 19884 |
| Sexual and reproductive health among men from 12 to 49 years | Sexual activity, family planning, sexual transmitted infections | 15962 |
| Anthropometry and blood pressure | Weight, height, and other anthropometric measures among subjects older than 10 years | 57727 |
| 24-hour dietary recall | Among participants from 1 to 60 years | 19932 |
| Biochemical analysis | Results of the biochemical analysis among subjects from 6 month to 60 years in 50% of households | 21482 |
